# Ruxolitinib in Myelofibrosis and Polycythemia Vera

**Published:** 2016-05-01

**Authors:** Leah Wolfe

**Affiliations:** Hematology Oncology Center Inc. – Pharmacy, Elyria, Ohio

Myelofibrosis (MF) and polycythemia vera (PV) are Philadelphia chromosome (BCR-ABL1)-negative subtypes of chronic myeloproliferative neoplasms (MPNs; Hudnall, 2012; Swaim, 2014). These rare hematologic malignancies share dysregulated signaling of Janus-associated kinase-signal transducer and activator of transcription (JAK-STAT) pathways essential for transduction of signals to the cell nucleus (Furqan, Mukhi, Lee, & Liu, 2013). As a result, perpetually active signaling causes hyperproliferation of hematopoietic stem cells, extramedullary hematopoiesis, and excess release of cytokines, proteins that cause chronic inflammation and systemic itching (Lowery, 2013).

In 2005, researchers discovered the *JAK2^V617F^* mutation, which is present in 50% to 60% of patients with MF and 95% of patients with PV (Stein et al., 2015). This significant finding helped pave the way for development of targeted therapies designed to inhibit overactive JAK signaling (Lowery, 2013). In 2011, the US Food and Drug Administration (FDA) approved ruxolitinib (Jakafi) as the first and only JAK inhibitor indicated for treatment of patients with intermediate- or high-risk MF, including primary MF, post-PV MF, and post-essential thrombocythemia (ET) MF (Stenger, 2012). In 2014, the drug was approved for patients with PV who have inadequate response to or intolerance of the drug hydroxyurea (Inman, 2014).

In MF, unchecked JAK signaling leads to abnormal blood counts, bone marrow fibrosis, and excess production of inflammatory cytokines (Swaim, 2014). As hematopoiesis is forced to the spleen, splenomegaly causes feeling of early satiety and abdominal pain (Lowery, 2013). Quality of life is often significantly diminished by an interrelated symptom burden, which includes fatigue, bone pain, pruritus, and night sweats.

Risk stratification of MF at diagnosis is determined using the International Prognostic Scoring System (IPSS), which assigns points based on five risk factors: age > 65 years, hemoglobin < 10 g/dL, leukocyte count > 25 × 10^9^/L, circulating blasts ≥ 1%, and presence of constitutional symptoms. Based on their score, patients are categorized as low-, intermediate-1, intermediate-2, or high-risk (Tefferi, 2013). Although it is possible for the disease to occur at any time, the median age at diagnosis is 65 years (Manea, 2014). Myelofibrosis can progress to acute myelogenous leukemia (Hudnall, 2012). The prevalence of MF in the United States is 4 to 6 per 100,000 population (Stein et al., 2015).

Typically diagnosed in older adults, PV is characterized by increased red cell production and may present elevated white cell and platelet counts (Hudnall, 2012). Patients experience increased blood viscosity and are at greater risk for thrombohemorrhagic events (Raedler, 2014b). Symptoms can include splenomegaly, fatigue, pruritus, and night sweats (Raedler, 2014a). The primary treatment goal is to minimize thrombosis. Therefore, patients are risk stratified based on age and thrombotic history (low to moderate risk: ≤ age 60 and no prior thrombosis; high risk: > age 60 and previous thrombosis; Tefferi & Barbui, 2015).

Treatment includes low-dose aspirin and phlebotomy, with a target hematocrit value of < 45% to reduce thrombotic complications. In addition, hydroxyurea is often prescribed when patients are classified as high risk (Monga & Devetten, 2010). Over a 10-year span, PV progresses to MF (considered secondary MF) and acute myelogenous leukemia at rates of approximately 10% and < 3%, respectively (Harrison & Vannucchi, 2012; Incyte, 2014b). Prevalence of PV in the United States is estimated to be 44 to 57 per 100,000 population (Stein et al., 2015).

## MECHANISM OF ACTION AND PHARMACOKINETICS

**Figure 1:**
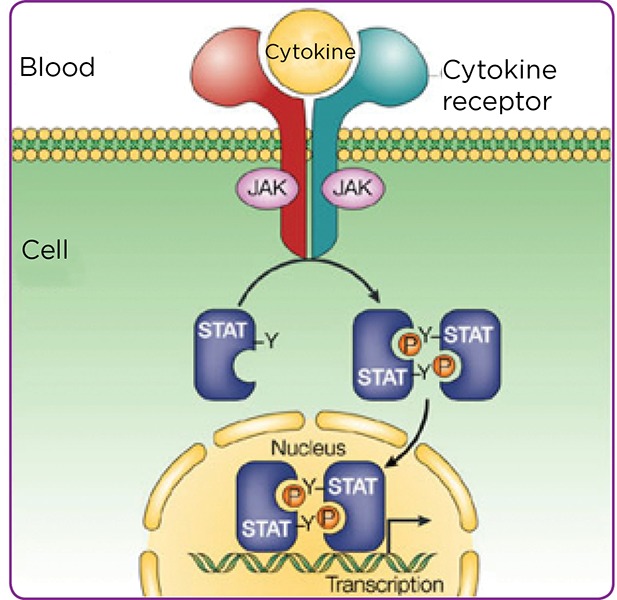
Overactive JAK-STAT pathway signaling: Mechanism of disease. JAKs are intracellular tyrosine kinases that relay extracellular signals via type I cytokine receptors (e.g., erythropoietin and thrombopoietin receptors in hematopoietic cells) to stimulate cell proliferation, differentiation, and survival as well as the production of proinflammatory cytokines. Adapted from The University of Texas MD Anderson Cancer Center (2015).

Ruxolitinib, a kinase inhibitor, down-regulates overactive signaling of JAK1 and JAK2, which are responsible for mediating signals of cytokines and growth factors necessary for hematopoiesis and immune function (see Figure 1). Though there is some overlap, JAK1 plays a role in mediating several proinflammatory cytokines, whereas JAK2 is primarily responsible for hematopoietic growth factors (Swaim, 2014).

Ruxolitinib is rapidly absorbed, achieving peak concentration within 1 to 2 hours of dosing, with bioavailability of at least 95% (Swaim, 2014). The drug has a volume of distribution of 72–75 L and is protein bound. Ruxolitinib is metabolized by the liver via CYP3A4 and eliminated primarily by the kidneys (74%). The mean elimination half-life is approximately 3 hours (5.8 hours for ruxolitinib and metabolites; Incyte Corporation, 2014a).

## CLINICAL TRIALS

**Myelofibrosis: COMFORT-I and COMFORT-II**

Ruxolitinib for intermediate-2 and high-risk MF was evaluated in two phase III clinical trials. COMFORT-I enrolled 309 patients in a double-blind, randomized, placebo-controlled study. Median age was 68 years, and median baseline spleen volumes were 2,598 cm³ (ruxolitinib) and 2,566 cm³ (placebo; normal is ≤ 300 cm³). The primary endpoint was proportion of patients who achieved ≥ 35% reduction in spleen volume by week 24. Secondary endpoints were durability of response, reduction in total symptom score, and overall survival (Verstovsek et al., 2012).

COMFORT-II was an open-label study of 219 patients who were randomized 2:1 to ruxolitinib or best available therapy (BAT) on a patient-by-patient basis. The median patient age was 66.5 years, and the median baseline spleen volumes were 2,208 cm³ (ruxolitinib) and 2,318 cm³ (BAT). The primary endpoint was proportion of patients achieving ≥ 35% reduction in spleen volume at week 48. The secondary endpoints included proportion of patients achieving a ≥ 35% reduction in spleen volume at week 24 and overall survival (Harrison et al., 2012; Cervantes et al., 2013).

**Figure 2:**
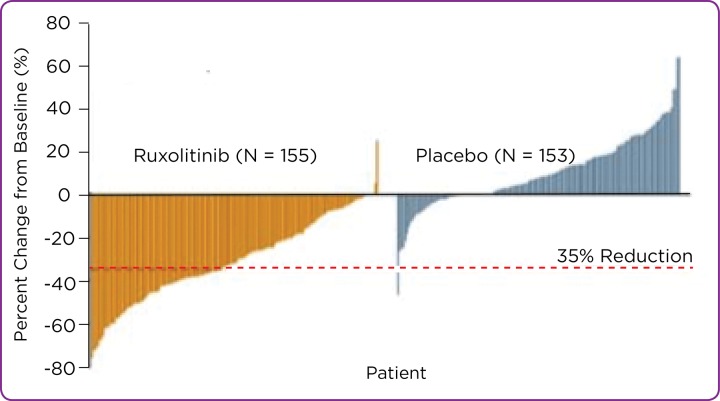
Percent change from baseline in spleen volume at week 24 or last observation for each patient in the study (COMFORT-I). Adapted from Verstovsek et al. (2012).

Both studies demonstrated that a statistically significant proportion of patients taking ruxolitinib achieved ≥ 35% reduction in spleen volume from baseline compared with placebo (COMFORT-I; see Figure 2) or BAT (COMFORT-II; Lowery, 2013). In the COMFORT-I trial, 46% of subjects taking ruxolitinib demonstrated ≥ 50% reduction in total symptom score vs. 5% receiving placebo (Verstovsek et al., 2012). Additionally, in the COMFORT-II trial, 28% in the ruxolitinib group achieved ≥ 35% reduction in spleen volume vs. 0% for those receiving BAT at week 48. The corresponding results at week 24 were 32% and 0% (Harrison et al., 2012).

**Figure 3:**
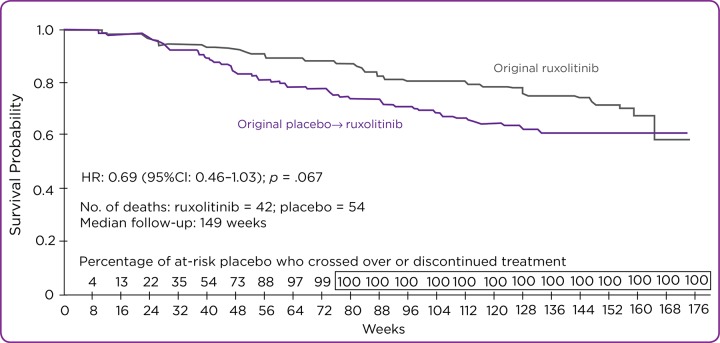
Overall survival: Kaplan-Meier curves by treatment group in COMFORT-I. HR = hazard ratio; CI = confidence interval. Adapted from Verstovsek et al. (2015a).

**Figure 4:**
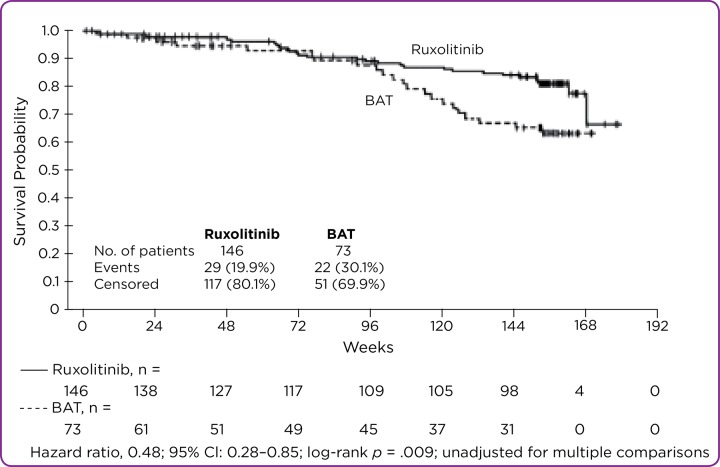
Overall survival: Kaplan-Meier curves by treatment group in COMFORT-II. BAT = best available treatment; CI = confidence interval. Adapted from Cervantes et al. (2015).

After 3 years of study, ruxolitinib demonstrated a measurable survival probability in both COMFORT-I and COMFORT-II, as shown in the Kaplan-Meier curves in Figures 3 and 4 (Verstovsek et al., 2015a; Cervantes et al., 2013).

**Polycythemia Vera: RESPONSE Trial**

The RESPONSE trial was an international, open-label, multicenter phase III trial randomizing 1:1 ruxolitinib to BAT. Participants included 222 phlebotomy-dependent PV patients with splenomegaly who were resistant to or intolerant of hydroxyurea. The primary endpoint was twofold: achievement of hematocrit control and spleen volume reduction from baseline of ≥ 35% at week 32. Secondary endpoints included proportion of patients who achieved the primary endpoint and maintained their response at week 48 and proportion of patients who had complete hematologic remission (as defined by study protocol) at week 32.

Results of primary and secondary endpoints are shown in Table 1. Additionally, 49% of the therapy group demonstrated at least a 50% reduction in total symptom score vs. 5% in the BAT group. Most patients receiving BAT crossed over to ruxolitinib at or immediately after week 32 (Vannucchi et al., 2015). As a result, the impact on overall survival could not be determined (Ignoffo, 2015).

**Table 1:**
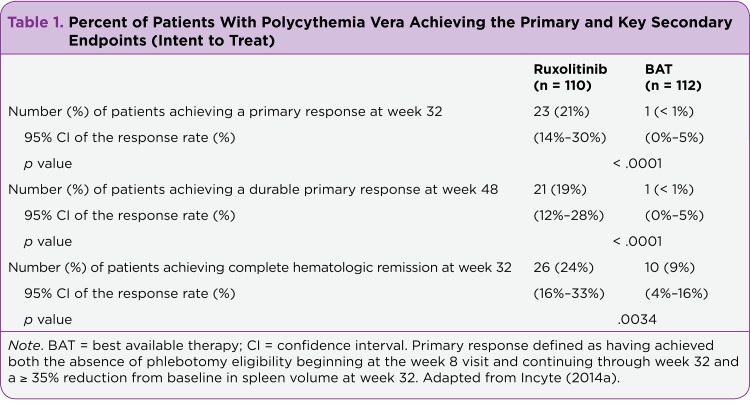
Percent of Patients With Polycythemia Vera Achieving the Primary and Key Secondary Endpoints (Intent to Treat)

## DOSING, MODIFICATIONS, AND SPECIAL POPULATIONS

Ruxolitinib is available through a limited pharmacy network (Swaim, 2014). The oral medication is administered by tablet twice daily in adult and geriatric populations. It has five strengths (5, 10, 15, 20, and 25 mg) and may be dissolved in 40 mL of water if administration via gastric tube (8 French or greater) is necessary (Lowery, 2013). Dosing is dependent on complete blood cell count (CBC) and platelet count, with modifications for patients with renal or hepatic impairment. Patients who miss a dose are directed to take the next dose at the regular time and must not take an additional dose. Ruxolitinib is stored at room temperature. It may be taken with or without food, but patients should be advised to avoid grapefruit juice. They should report all over-the-counter, herbal, or dietary supplements prior to the start of ruxolitinib. If patients require hospitalization, they should take their medication with them (Incyte Corporation, 2014a).

**Dosing in Intermediate- or High-Risk Myelofibrosis**

Dosing for patients with MF is based on platelets. A baseline CBC and platelet count is necessary prior to the start of treatment. Blood work should be repeated every 2 to 4 weeks until doses are stabilized and continued thereafter as clinically indicated (Incyte Corporation, 2014a; See Table 2).

**Table 2:**
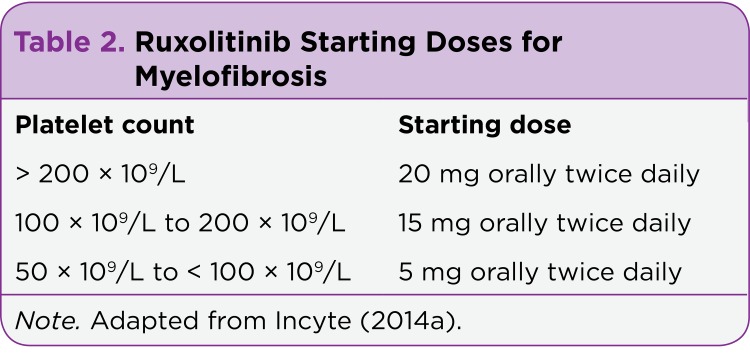
Ruxolitinib Starting Doses for Myelofibrosis

Dosing modification may be indicated for patients with insufficient response or hematologic toxicity. Treatment should be interrupted in cases where bleeding requires medical intervention and may resume at the prior or lower dose once the bleed has been resolved. Additionally, treatment should be interrupted if platelet count is < 50 × 10^9^/L or absolute neutrophil count (ANC) is < 0.5 × 10^9^/L. Therapy may be resumed after the platelet count recovers to > 50 × 10^9^/L and ANC > 0.75 × 10^9^/L (Incyte, 2014a; See Table 3).

**Table 3:**
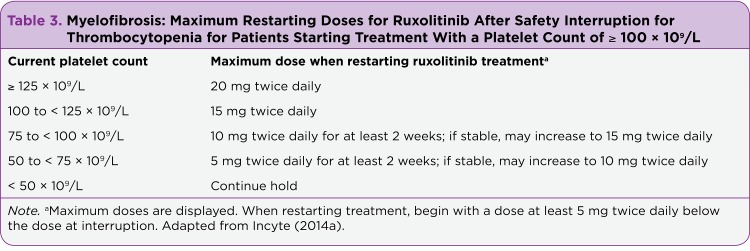
Myelofibrosis: Maximum Restarting Doses for Ruxolitinib After Safety Interruption for Thrombocytopenia for Patients Starting Treatment With a Platelet Count of ≥ 100 × 10^9^/L

**Dosing in Polycythemia Vera**

Before prescribing ruxolitinib for patients with PV, a baseline CBC and platelet count is necessary. For PV, the recommended starting dose of ruxolitinib is 10 mg/twice daily and may be titrated based on safety and efficacy. Bloodwork should be repeated every 2 to 4 weeks until doses are stabilized and thereafter as clinically indicated. Dose reductions should be considered for hemoglobin and platelet count decrease (Incyte Corporation, 2014a; See Table 4).

**Table 4:**
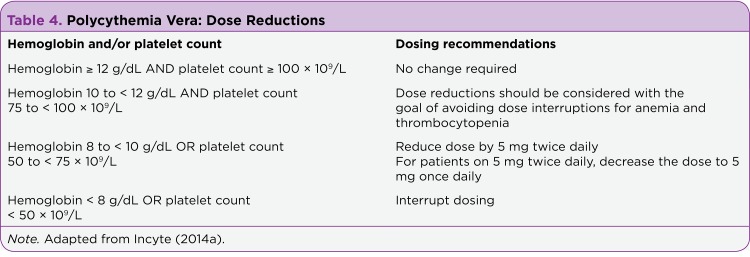
Polycythemia Vera: Dose Reductions

**Treatment Interruption and Restarting**

Treatment should be interrupted for hemoglobin (Hb) < 8 g/dL, platelet count < 50 × 10^9^/L, or ANC < 1.0 × 10^9^/L. After hematologic levels recover, doses may be restarted as follows, using the most severe category of a patient’s hemoglobin, platelet count, or ANC abnormality to determine the maximum restarting dose (Incyte Corporation, 2014a; See Table 5).

**Table 5:**
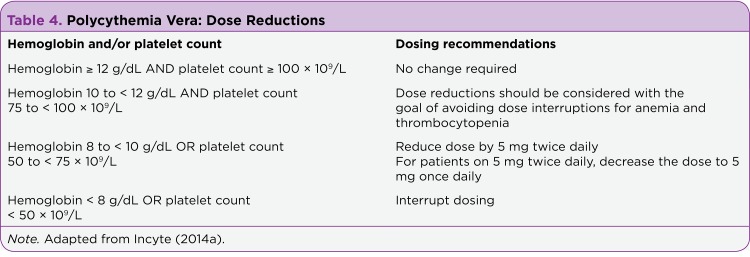
Polycythemia Vera: Restarting Doses for Ruxolitinib After Safety Interruption for Hematologic Parameter(s)

In both MF and PV, for complete information on dose modifications, as well as dosing for patients with renal or hepatic impairment, practitioners should refer to the full prescribing information available at http://www.jakafi.com/pdf/prescribing-information.pdf.

**Drug Interactions and Special Populations**

There are no contraindications for ruxolitinib. Dose modification may be indicated when it is prescribed concomitantly with CYP3A4 inhibitors or inducers and/or fluconazole doses of less than or equal to 200 mg daily. Avoid the use of fluconazole doses greater than 200 mg daily concomitantly with ruxolitinib. Check drug interactions before prescribing. Ruxolitinib is a Category C drug in pregnancy, should not be taken by nursing mothers, and has not been studied in pediatrics. Patients with renal impairment on dialysis should be advised to take their dose after dialysis. Ruxolitinib should be avoided in patients with end-stage renal disease who are not on dialysis (Incyte Corporation, 2014a).

## WARNINGS AND PRECAUTIONS

Treatment may cause thrombocytopenia, anemia, and neutropenia, which are often managed by dose modification and/or blood transfusion (Lowery, 2013). Monitor CBC levels throughout treatment and advise patients to report bleeding or bruising. Ruxolitinib may increase the risk of nonmelanoma skin cancers. Patients should be advised to monitor skin changes (Incyte Corporation, 2014a).

Delay the start of ruxolitinib for patients with active infection. Patients should be monitored for and educated about tuberculosis, progressive multifocal leukoencephalopathy, and herpes zoster. Patients should report potential signs of infection, including chills, aches, fever, nausea/vomiting, or skin rash/blisters (Incyte Corporation, 2014a).

**Adverse Reactions, Treatment Discontinuation, and Symptom Exacerbation**

During investigational trials, the most prevalent hematologic adverse effects for MF patients were thrombocytopenia and anemia, both related to dosing. The most common nonhematologic symptoms reported by COMFORT-I ruxolitinib patients were ecchymosis, dizziness, and headache; COMFORT-II ruxolitinib patients most frequently experienced diarrhea and abdominal pain (Harrison et al., 2012; Verstovsek et al., 2012).

Ruxolitinib was generally well tolerated by patients in the RESPONSE trial. At a median follow-up of 111 weeks, 83% of patients were still receiving treatment (Verstovsek et al., 2015b). The most common adverse reactions were anemia and thrombocytopenia (Ignoffo, 2015). Additionally, > 10% of patients reported headache, diarrhea, fatigue, pruritus, dizziness, muscle spasm, dyspnea, and/or abdominal pain (Vannucchi et al., 2015).

Reasons for discontinuation of treatment have included loss/lack of response, disease progression, toxicity, and patient or physician choice. Patients who stop ruxolitinib may experience an acute relapse of symptoms and splenomegaly, sometimes requiring emergency intervention (Tefferi & Pardanani, 2011). Prescribers should caution against interruption or discontinuation of ruxolitinib without medical consultation. Except in cases of thrombocytopenia or neutropenia, doses should be gradually tapered (Swaim, 2014).

## IMPLICATIONS FOR ADVANCED PRACTITIONERS

Myelofibrosis and polycythemia vera are rare clonal disorders of hematopoietic stem cells characterized by varied pathogenesis, debilitating symptom burden, and high mortality rates (Harrison & Vannucchi, 2012). Ruxolitinib demonstrates a noteworthy evolution in treatment options by offering patients the hope of living longer and experiencing significant improvement in quality of life. Currently, National Comprehensive Cancer Network Guidelines for MPNs are in the planning stage (Stein, O’Brien, Greenberg, & Mesa, 2015). Advanced practitioners in oncology are part of a crucial team, along with oncologists, hematologists, pharmacists, and physician assistants, ensuring that patients receive the monitoring, counseling, and support necessary to achieve optimal medication adherence and symptom management throughout treatment.

**Acknowledgments**

The author would like to acknowledge Kelley Gray, MA, BS, for writing and editing support throughout the production of this manuscript.
